# Supplementary material for: The transcription factor CBFB suppresses breast cancer through orchestrating translation and transcription
Source: Nat Commun. 2019 May 6;10:2071. doi: 10.1038/s41467-019-10102-6 (PMC6502810; doi:10.1038/s41467-019-10102-6)
Supplement: Supplementary file 1 — Supplementary Information [file 41467_2019_10102_MOESM1_ESM.pdf]

Supplementary information

The transcription factor CBFB suppresses breast cancer through orchestrating translation and transcription

Malik, et al.

Supplementary Fig. 1

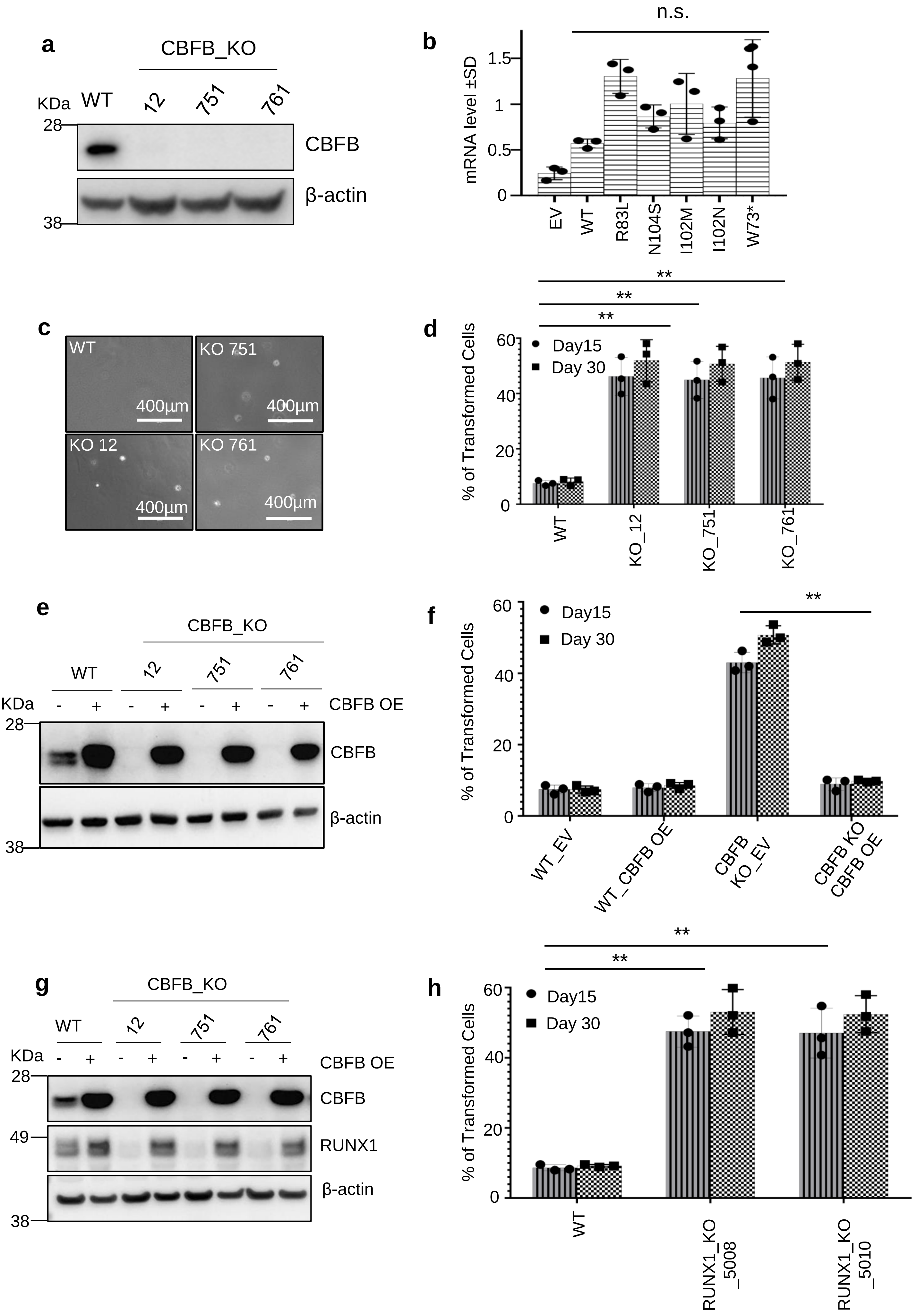

Supplementary Fig. 1. CBFB and RUNX1 have tumor suppressive roles in breast cancer. (**a**) Immunoblotting (IB) showing deletion of CBFB by CRISPR in MCF10A cells. (**b**) Realtime PCR showing mRNA levels of *CBFB* WT and mutants. Error bars in SEM, n=3 (biological); n.s., p value >0.05 (WT Vs CBFB Mutants). (**c**) and (**d**), Anchorage independent assays showing the transformation of CBFB_KO MCF10A cells. WT and three KO clones, KO_12, KO_751, and KO_761, were shown. (**c**) Representative images; (**d**) Percentage of transformed cells judged by number of colonies. See Supplemental Methods for the calculations of percentage of transformed cells and colony size. n=3(biological); two asterisks, p value <0.01 (WT Vs CBFB KOs) (**e**) IB showing overexpression (OE) of CBFB in CBFB KO clones. (**f**) Anchorage independent growth assay of CBFB WT and KO clones transduced with lentivirus expressing an empty vector (EV) or CBFB. Error bars in SEM, n=3 (Biological); two asterisks, p value<0.01(CBFB KO_EV Vs CBFB KO_CBFB OE). (**g**) IB showing the effect of CBFB OE on the expression of RUNX1 in CBFB KO MCF10A cells. (**h**) Anchorage independent growth assay of WT and RUNX1 KO clones. Percentages of transformed cells were shown. Error bars in SEM, n=3 (biological); p value <0.01 (WT versus RUNX1 KOs). p values are calculated with two-tailed t-test.

Supplementary Fig. 2

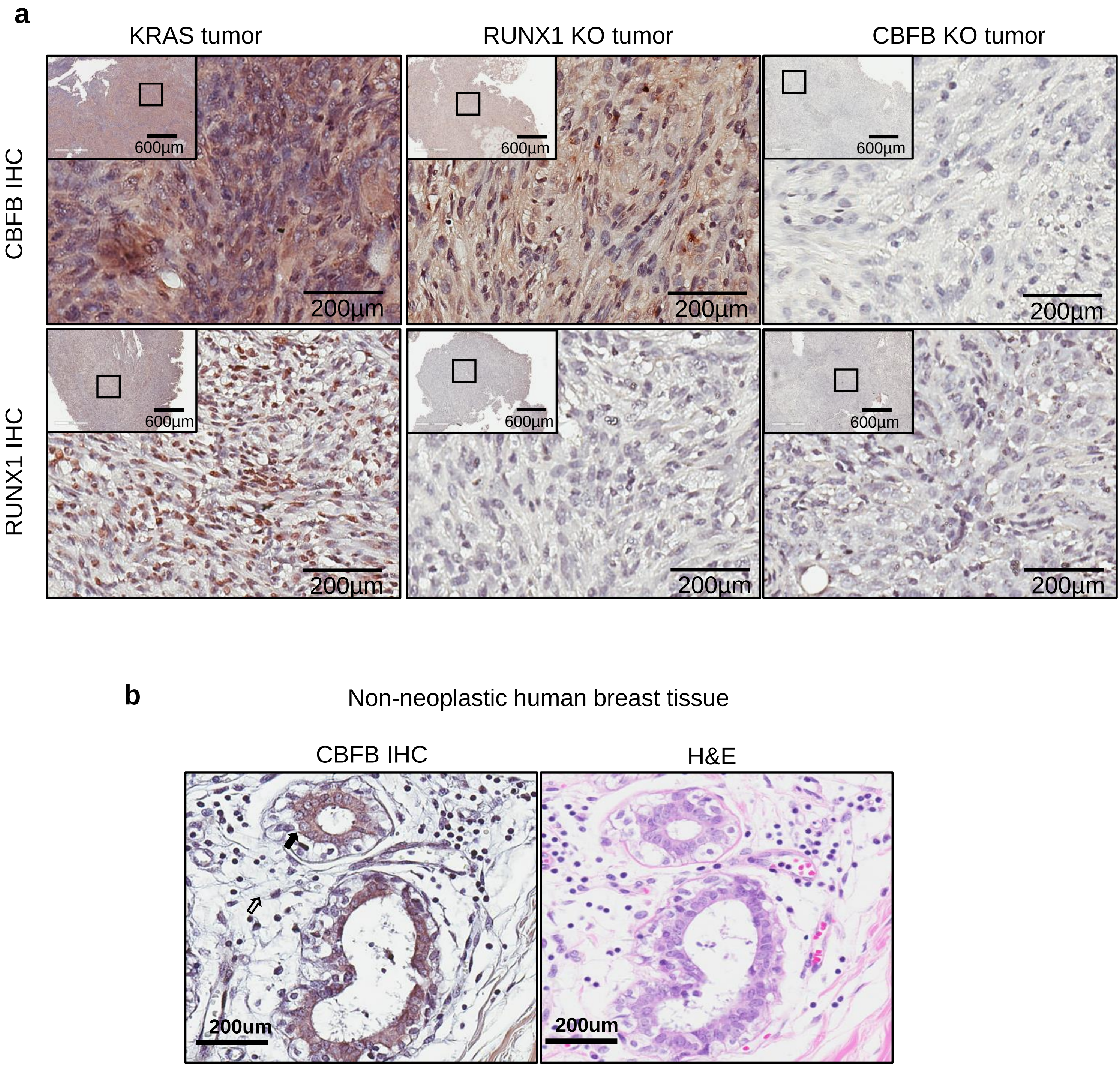

Supplementary Fig. 2. Subcellular localization of CBFB and RUNX1 in breast tumors and non-neoplastic human breast tissue. (a) Immunohistochemistry (IHC) staining of CBFB and RUNX1 in tumors derived from KRAS transformed, RUNX1 KO, and CBFB KO MCF10A cells. (**b**) IHC of CBFB in non-neoplastic human breast tissue. The solid arrow indicates epithelial ductal cells with predominantly cytoplasmic CBFB staining; the hollow arrow a lymphocyte with nuclear CBFB staining.

Supplementary Fig. 3

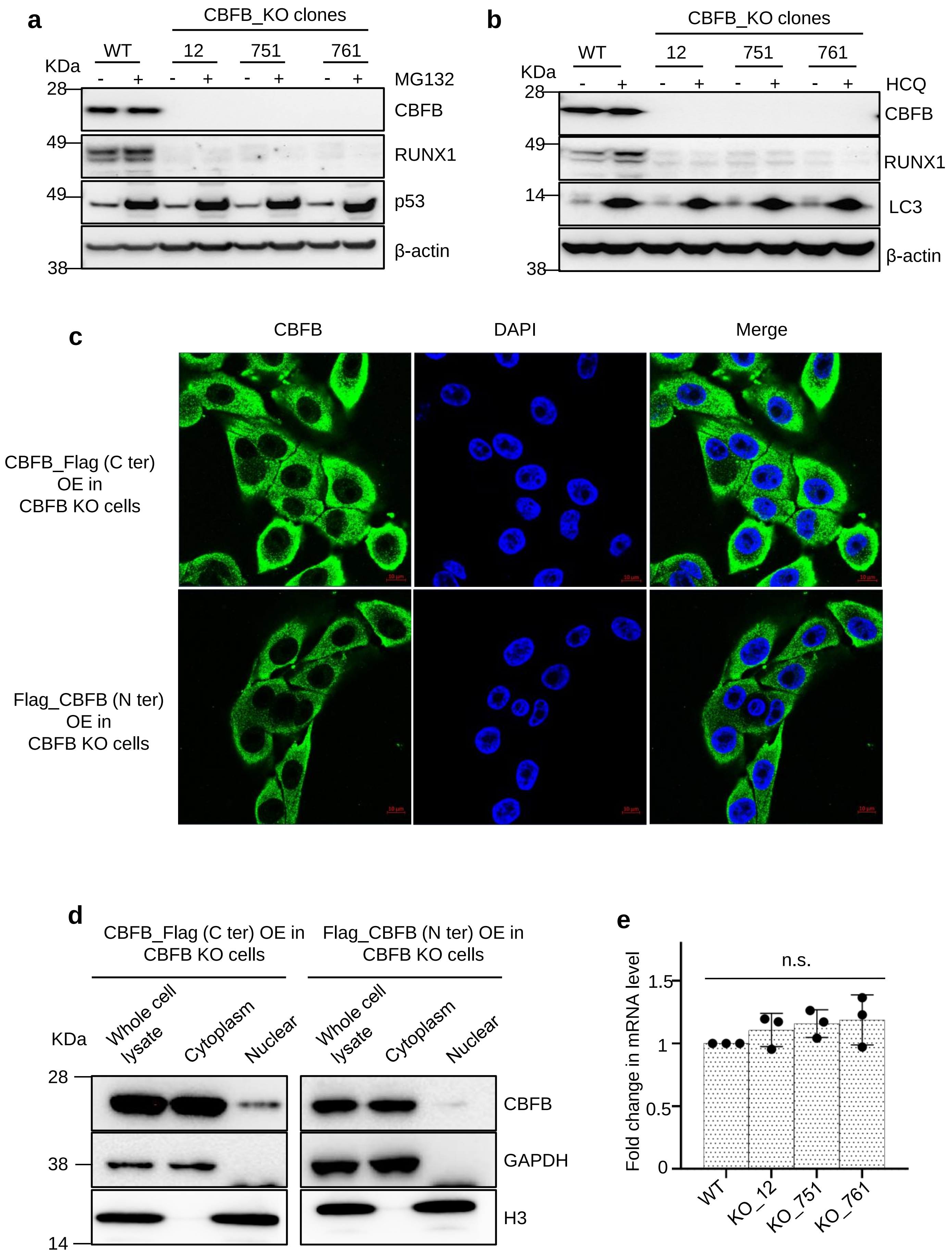

Supplementary Fig. 3

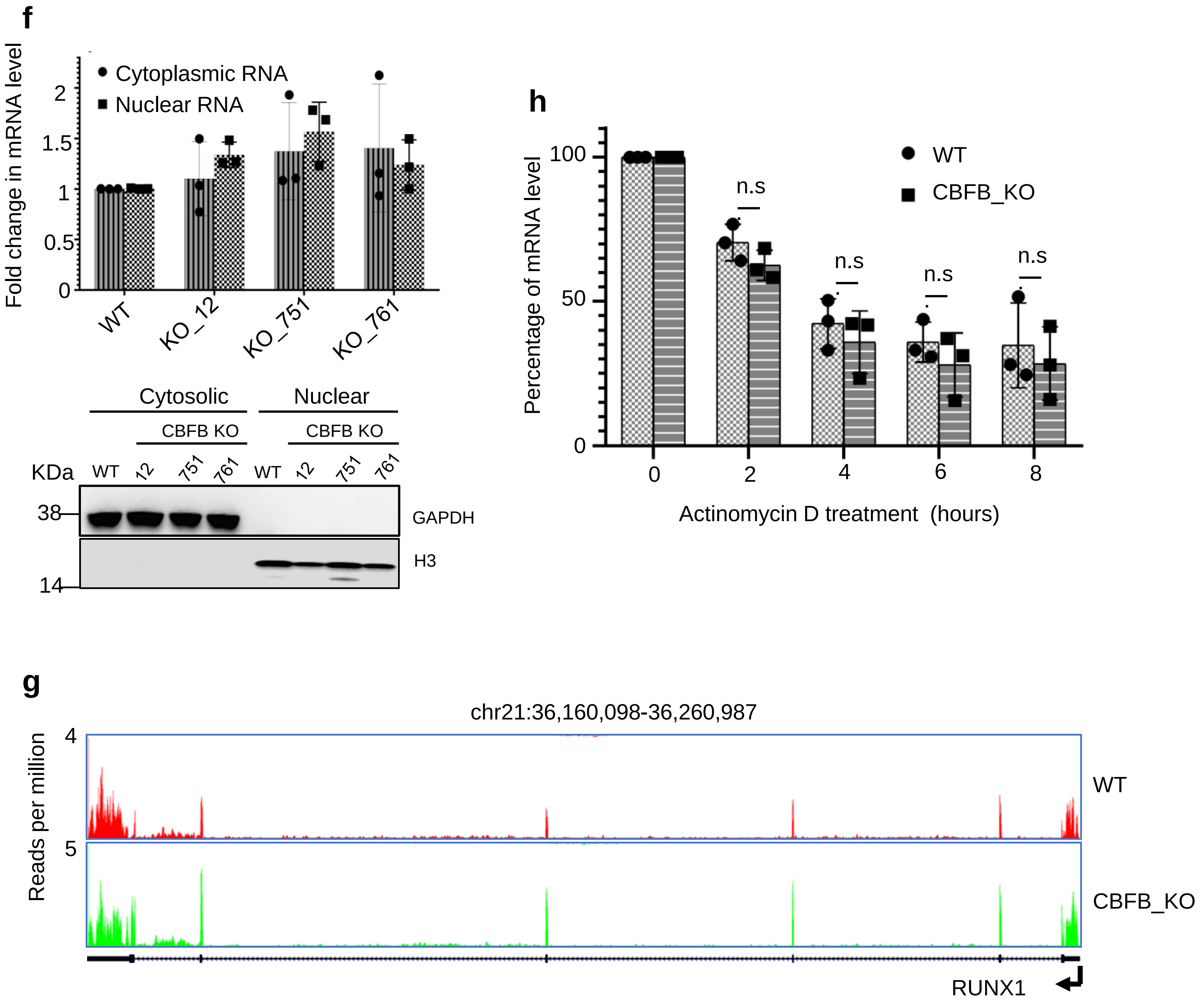

Supplementary Fig. 3. CBFB does not regulate the degradation of RUNX1 protein, steady-state levels, nucleus/cytoplasm distribution, degradation, and splicing of *RUNX1* mRNA and is mainly localized in the cytoplasm of breast cells. (**a**) IB showing the treatment of MG132 (proteasome inhibitor) in CBFB WT and KO clones. p53 IB was performed as a positive control for MG132 treatment. (**b)** IB of hydroxychloroquine (HCQ) treatment in CBFB WT and KO clones. LC3, a positive control for lysosomal degradation inhibition. IF (**c**) and IB (**d**) showing subcellular distribution of CBFB fused to an amino (N-ter) or carboxyl terminal (C-ter) Flag tag that was stably expressed in CBFB KO MCF10A cells. (**e**) Realtime PCR showing the steady-state levels of *RUNX1* mRNA in CBFB WT and KO clones. Error bars are SEM; n=3 (Biological); n.s, p value >0.05 (WT versus KO clones). **(f)** Realtime PCR showing the subcellular distribution of *RUNX1* mRNA in the cytoplasm and nucleus of CBFB WT and KO clones. Error bars are SEM; n=3 (Biological); p value >0.05 (WT versus KO clones). GAPDH, a marker for cytoplasm; histone H3, a marker for nuclei. (**g**) RNAseq showing the splicing of the *RUNX1* gene in CBFB WT and KO clones. Coordinates of the *RUNX1* gene was shown on top. Arrow on the bottom indicates the transcription direction. (**h**) Realtime PCR showing the *RUNX1* mRNA levels in WT and CBFB KO MCF10A cells upon treatment with actinomycin D (1 µg/ml) for different time periods. Error bars are SEM; n=3 (Biological); n.s., p value >0.05 (WT versus KO clones). All p values are calculated with two-tailed t-test.

Supplementary Fig. 4

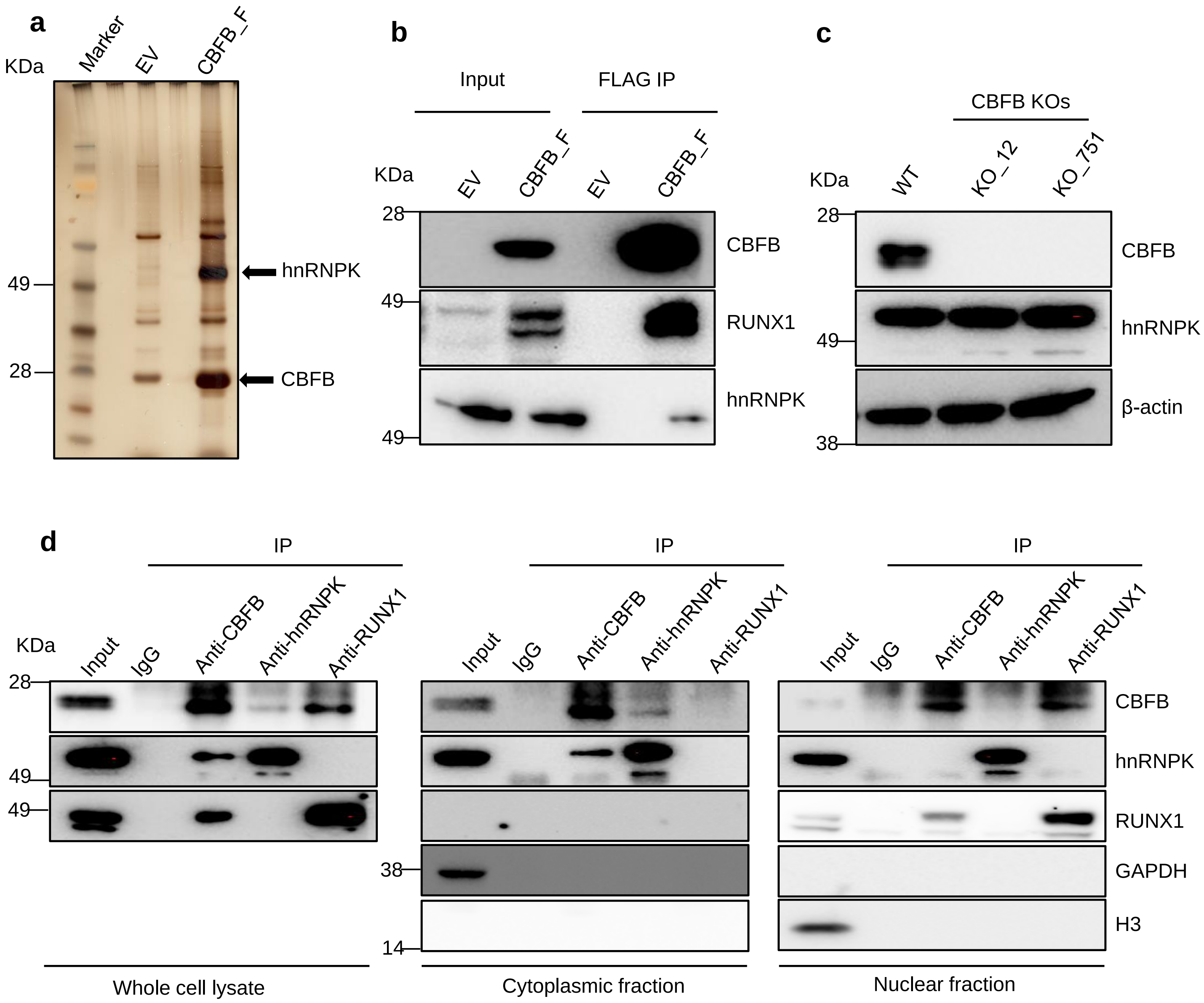

Supplementary Fig. 4

e

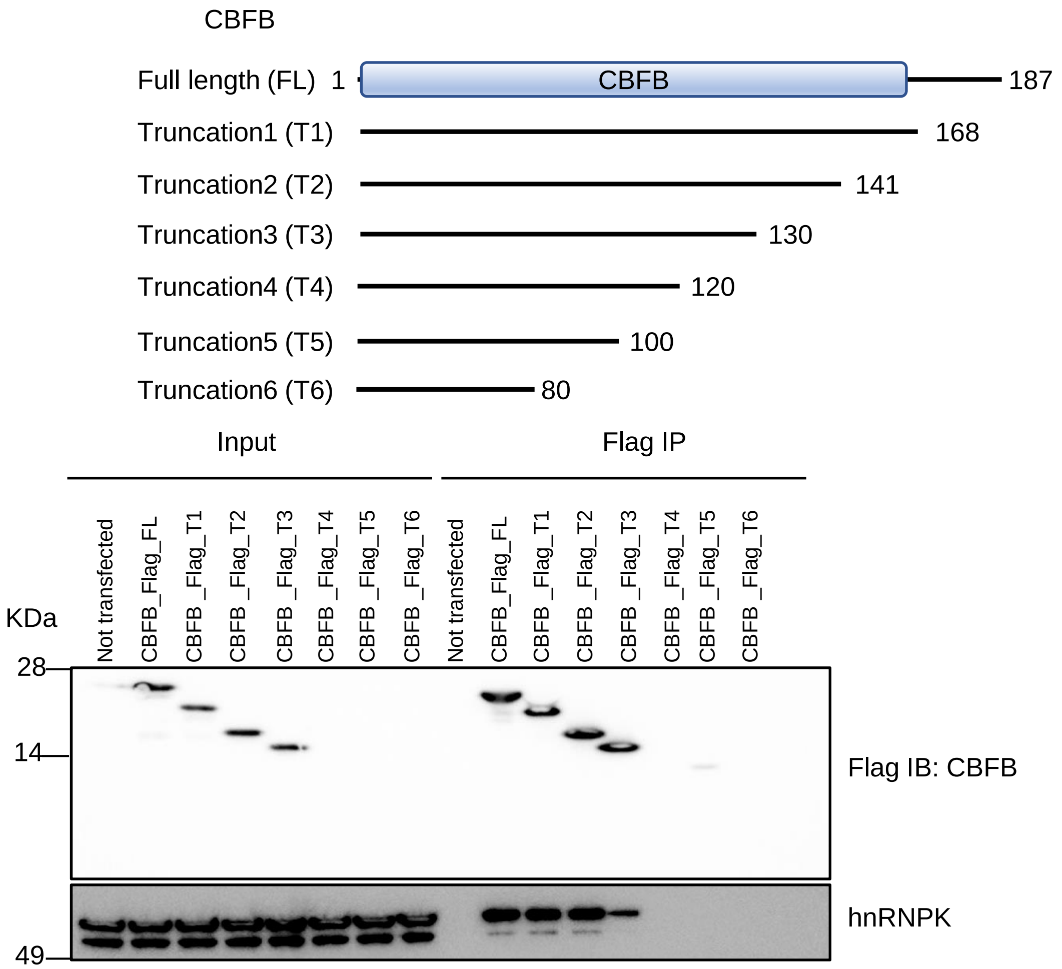

f

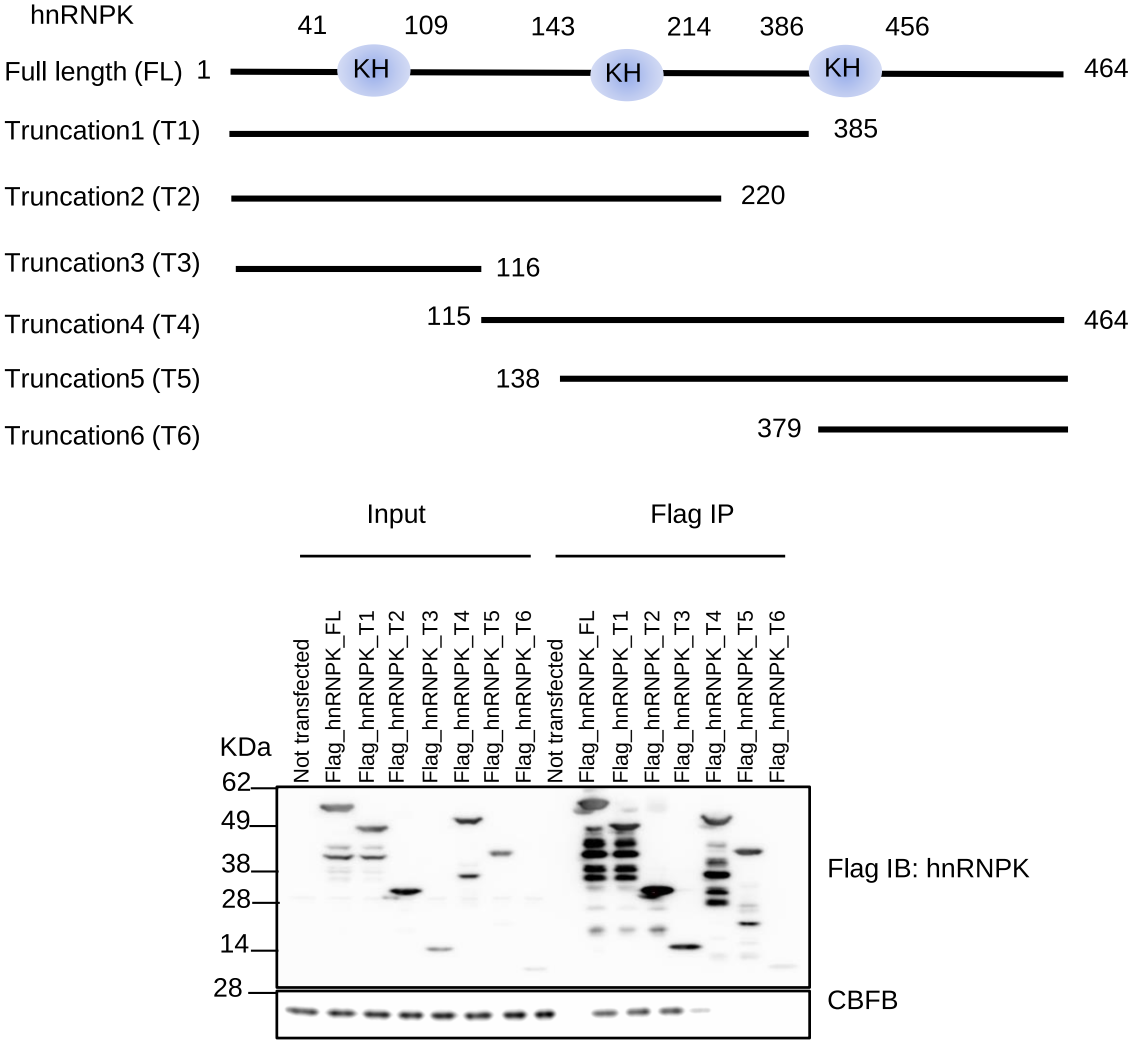

Supplementary Fig. 4. CBFB interacts with RNA binding protein hnRNPK. (**a**) Silver staining of Flag IP in CBFB KO MCF10A cells stably transduced with empty vector (EV) and CBFB_F (a Flag tag fused to the carboxyl terminus). **(b)** IB validation of CBFB_F and hnRNPK interaction. (**c**) IB showing expression of hnRNPK in CBFB WT and KO MCF10A cells. (**d**) Co-IP followed by IB showing interaction of endogenous CBFB, RUNX1, and hnRNPK in whole cell lysate, cytoplasmic and nuclear fractions of MCF10A cells. 10 ug/ml RNase A were added in the co-IP lysate. (**e**) Flag IP followed by Flag IB showing the interaction of transiently transfected CBFB_Flag truncated variants with endogenous hnRNPK in CBFB KO 293T cells. Full length CBFB, 187 amino acids (AA), truncation 1(T1, 1-168AA), truncation 2 (T2, 1-141 AA), truncation 3 (T3, 1-130AA), truncation 4 (T4, 1-120AA), truncation 5 (T5, 1-100 AA), and truncation 6 (T6, 1-80 AA). Shown in light blue is the CBFB domain. (**f**) Flag IP followed by Flag IB showing the interaction of transiently transfected Flag_hnRNPK truncated variants with endogenous CBFB in 293T cells. Full length hnRNPK, 464 amino acids (AA), truncation 1(T1, 1-385 AA), truncation 2(T2, 1-220AA), truncation 3 (T3, 1-116 AA), truncation 4 (T4, 115-464AA), truncation 5 (T5, 138-464AA), and truncation 6 (T6, 379-464 AA). Shown in light blue are the KH domains.

Supplementary Fig. 5

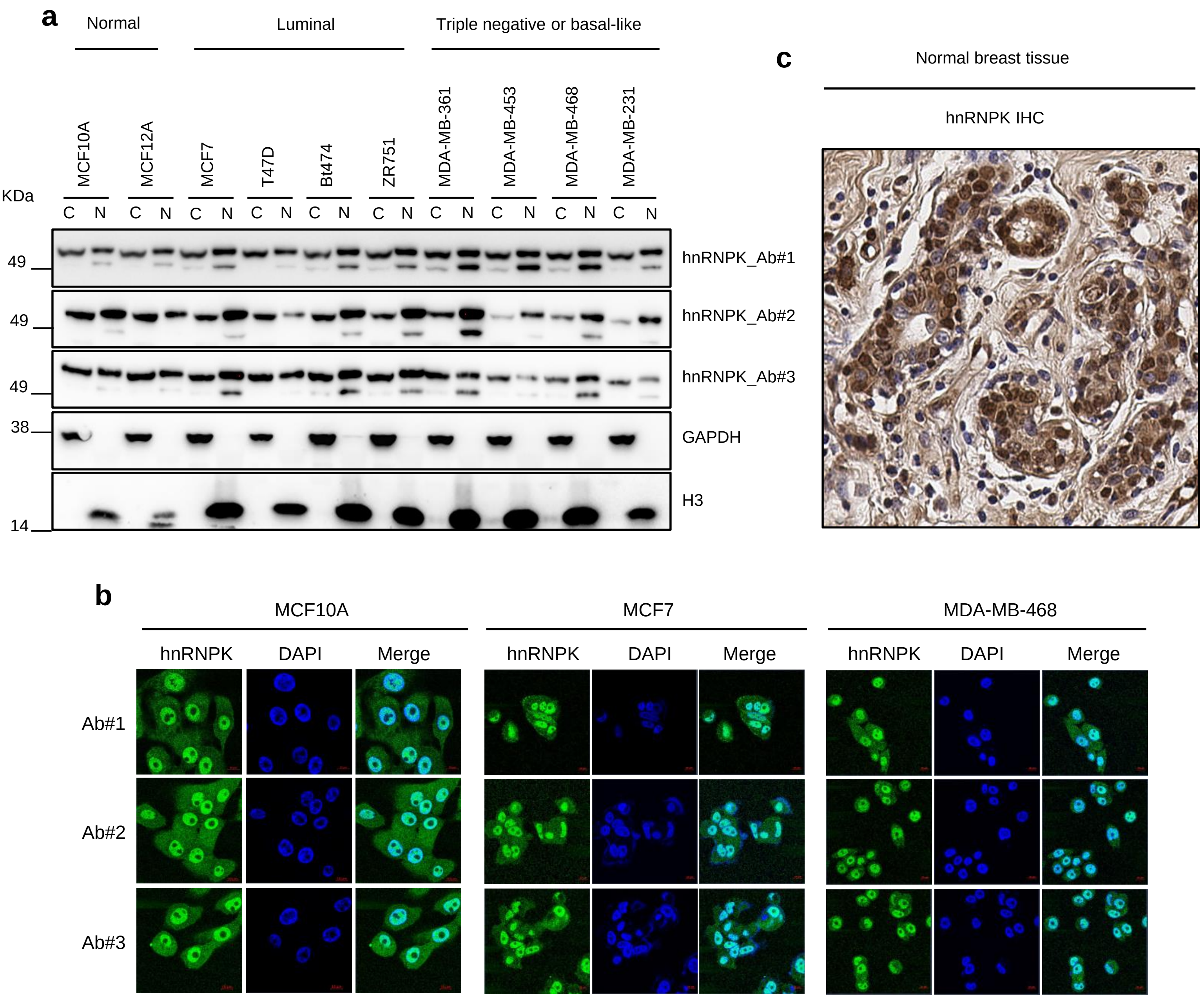

Supplementary Figure 5. hnRNPK is localized in both cytoplasm and nucleus of breast cells. (**a**) Immunoblotting showing the subcellular localization of hnRNPK. Different hnRNPK antibodies were: Ab#1(A300-674A), Ab#2 (A300-675A), and Ab#3 (A300-676A). (**b**) Immunofluorescence showing the subcellular localization of hnRNPK in MCF10A, MCF7, and MDA-MB-468 cells using three different hnRNPK antibodies. (**c**) IHC showing the subcellular localization of hnRNPK in normal breast tissue using Ab#1**.**

Supplementary Fig. 6

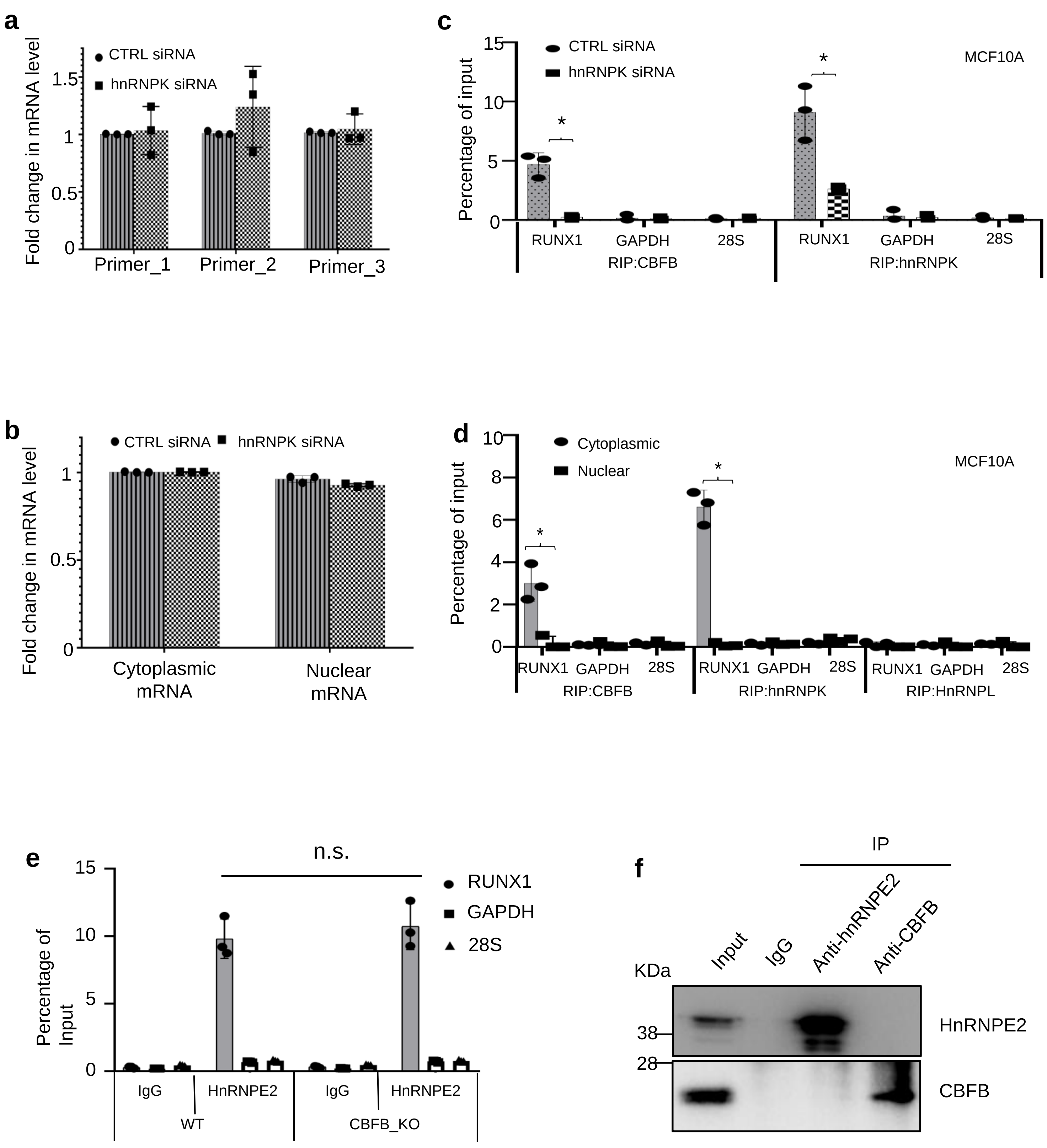

Supplementary Fig. 6

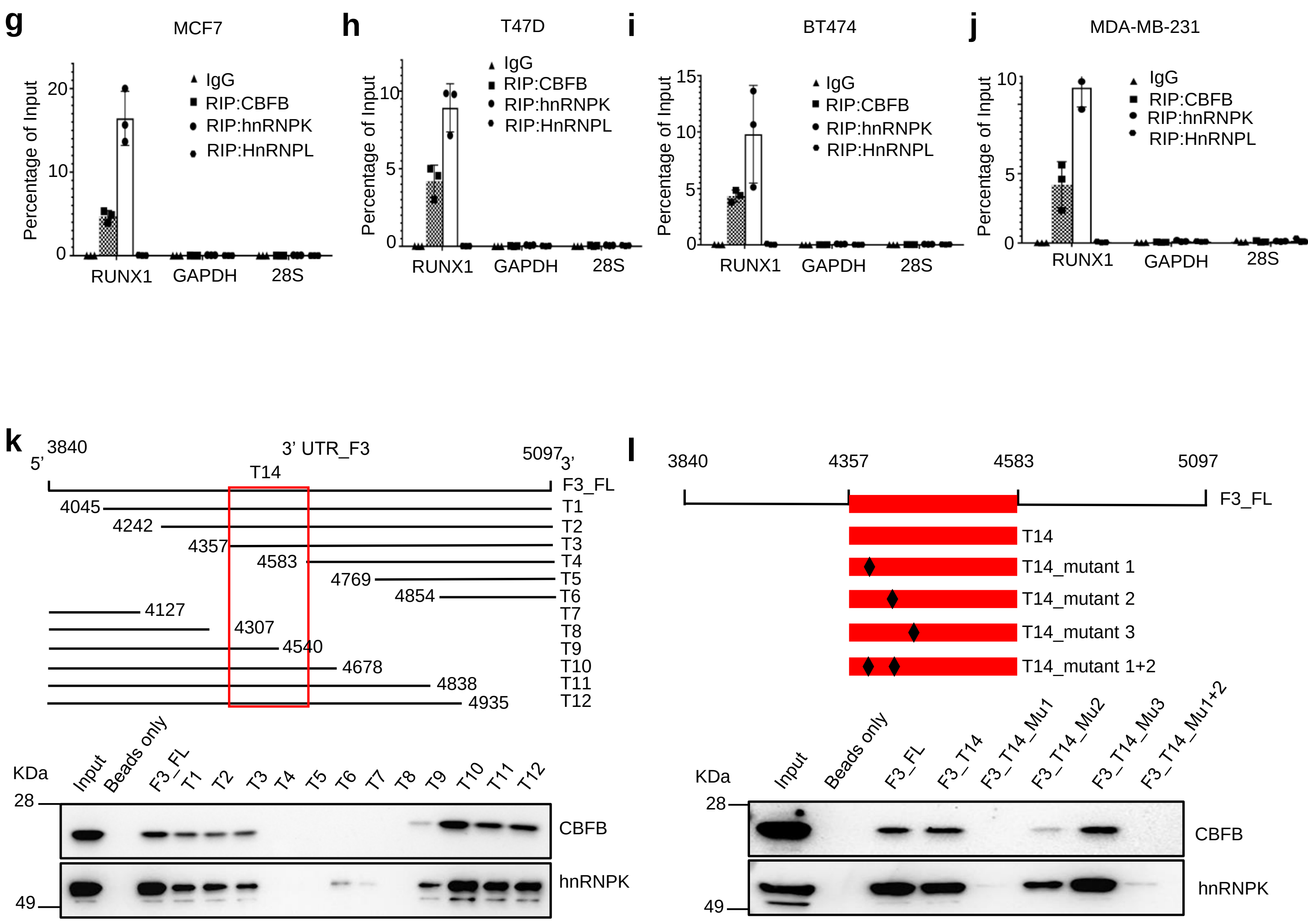

Supplementary Fig. 6. CBFB and hnRNPK bind to the 3' UTR of the *RUNX1* gene. (**a**) Realtime PCR measuring *RUNX1* mRNA in MCF10A cells transfected with control (CTRL) or hnRNPK siRNA. Error bars are SEM; n=3 (biological); p value >0.05. (**b**) Realtime PCR measuring *RUNX1* mRNA in the cytoplasmic and nuclear fractions of MCF10A cells transfected with control (CTRL) or hnRNPK siRNA. Error bars are SEM; n=3 (biological); p value >0.05 (cytosolic Vs nuclear *RUNX1* mRNA). (**c**) RIP of CBFB and hnRNPK in MCF10A cells transfected with control (CTRL) or hnRNPK siRNA. Error bars are SEM, n=3 (biological); asterisk, p<0.05. (**d**) Realtime PCR measuring *RUNX1* mRNA in RIP of CBFB, hnRNPK, and hnRNPL (negative control) in the cytoplasmic and nuclear fractions of MCF10A cells. Error bars are SEM; n=3 (biological); asterisk, p< 0.05 (RIP CBFB & hnRNPK in cytoplasm versus nuclear fractions). **(e)** RIP with IgG and hnRNPE2 antibody in WT and CBFB KO MCF10A cells. Error bars are SEM, n=3 (Biological); n.s., p value >0.05 (RIP of HnRNPE2 in WT versus CBFB KO cells). p values are calculated with two-tailed t-test. **(f)** Co-IP followed by CBFB IB showing interaction of endogenous CBFB and hnRNPE2 in MCF10A cells. (**g-j**) Realtime PCR measuring *RUNX1* and *GAPDH* mRNAs and *28s* ribosomal RNA in RIP of IgG (negative control), CBFB, hnRNPK, hnRNPL (negative control) in MCF7, T47D, BT474, and MDA-MB-231 cells. Error bars are SEM; n=3 (biological); p<0.05 (RIP_CBFB/hnRNPK versus IgG) (**k**) Mapping the binding region of hnRNPK and CBFB in F3 (fragment 3) of the 3' UTR of *RUNX1* mRNA. T1-12, truncated regions of F3 of 3' UTR of RUNX1 mRNA. Red square showing the binding region of CBFB and hnRNPK. (**l**) Site directed mutagenesis to determine the element required for hnRNPK and CBFB binding. Diamonds in the red thick line indicate poly-C tracts, which were changed to adenosine or thymidine. Mu, mutant.

Supplementary Fig. 7

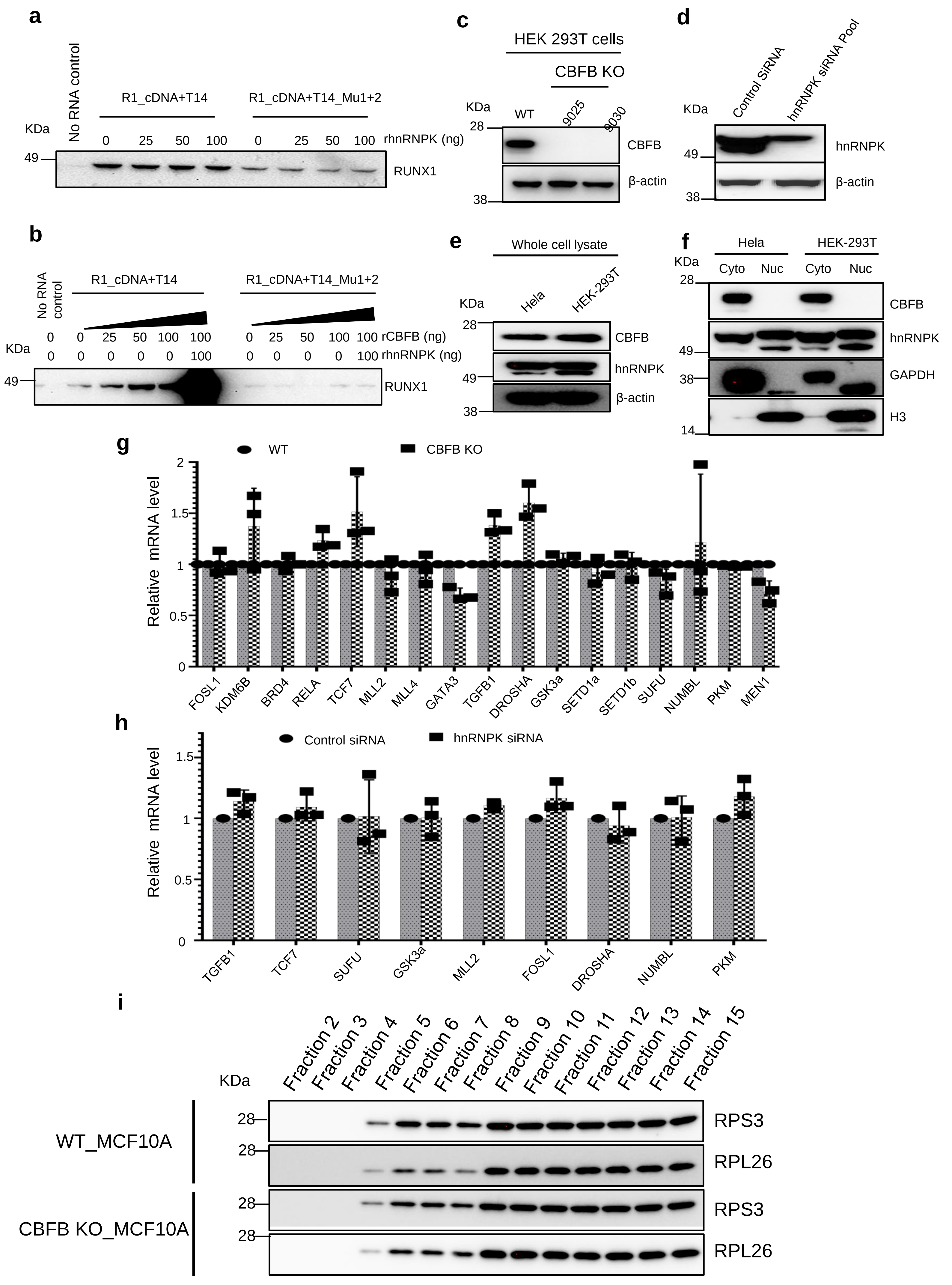

Supplementary Fig. 7. CBFB regulates translation. (**a**) Effect of recombinant hnRNPK protein on the translation of RUNX1. (**b**) Effect of recombinant CBFB along with recombinant hnRNPK protein on RUNX1 translation. (**c**) IB showing CBFB_KO in 293T cells. (**d**) IB showing hnRNPK knockdown (KD) in 293T cells. (**e**) IB showing the expression of CBFB and hnRNPK in Hela and 293T cells. **(f)** IB showing subcellular distribution of CBFB and hnRNPK in Hela and 293T cells. **(g)** RNAseq data showing the effect of CBFB deletion (CBFB_KO) on the expression of a number of CBFB/hnRNPK bound transcripts. The value of WT was normalized to 1. Error bars are in SEM, n=3(biological); p value >0.05. **(h)** Real time PCR data showing the effect of hnRNPK knockdown (hnRNPK_KD) on the expression of a number of CBFB/hnRNPK bound transcripts. Error bars are in SEM, n=3 (biological); p value > 0.05. **(i)** IB showing the presence of ribosome marker proteins (RPS3 and RPL26) in each fraction of WT and CBFB KO MCF10A cells.

Supplementary Fig. 8

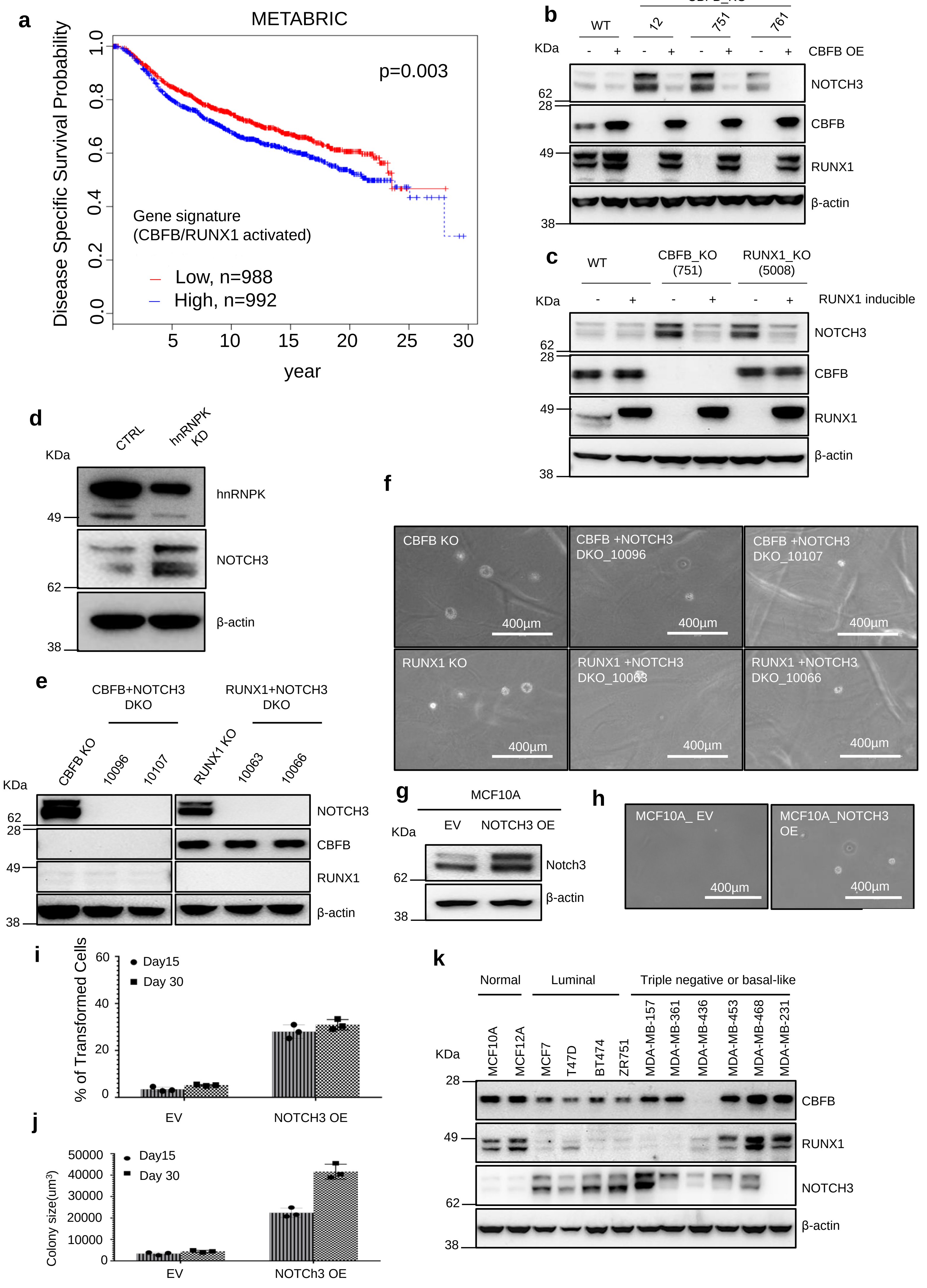

Supplementary Fig. 8. The role of CBFB in transcription regulation is also critical for its tumor suppressive function in breast cancer. (**a**) Kaplan Meier curve of patients in METABRIC stratified by a gene signature containing the top 10 genes activated by CBFB and RUNX1 in MCF10A cells. (**b**) IB showing the reversal of NOTCH3 upregulation in CBFB_KO clones upon overexpression (OE) of CBFB. (**c**) IB showing the reversal of NOTCH3 upregulation in CBFB KO clones upon OE of RUNX1. **(d)** IB showing the expression of NOTCH3 upon knockdown of hnRNPK in MCF10A cells. (**e**) IB showing the double knockout (DKO) of CBFB and NOTCH3 (CBFB+NOTCH3) or RUNX1 and NOTCH3 (RUNX1+NOTCH3). (**f**) Representative images of anchorage independent growth assays of CBFB_KO, RUNX1_KO and double knockout of CBFB+NOTCH3 and RUNX1+NOTCH3. n=3 (biological); p value <0.01 (EV Vs Notch3 OE).(**g**) IB showing exogenously expressed NOTCH3 in parental MCF10A cells. (**h**-**j**) Representative images, percentage of and size of colonies of anchorage independent assay of MCF10A cells transduced with lentiviruses expressing no cDNA or NOTCH3. Error bars are SEM; n=3 (biological); p value<0.01(EV Vs Notch3 OE). (**k**) IB showing expression of CBFB, RUNX1, and the NICD of NOTCH3 in multiple breast cells.

Supplementary Fig. 9

a

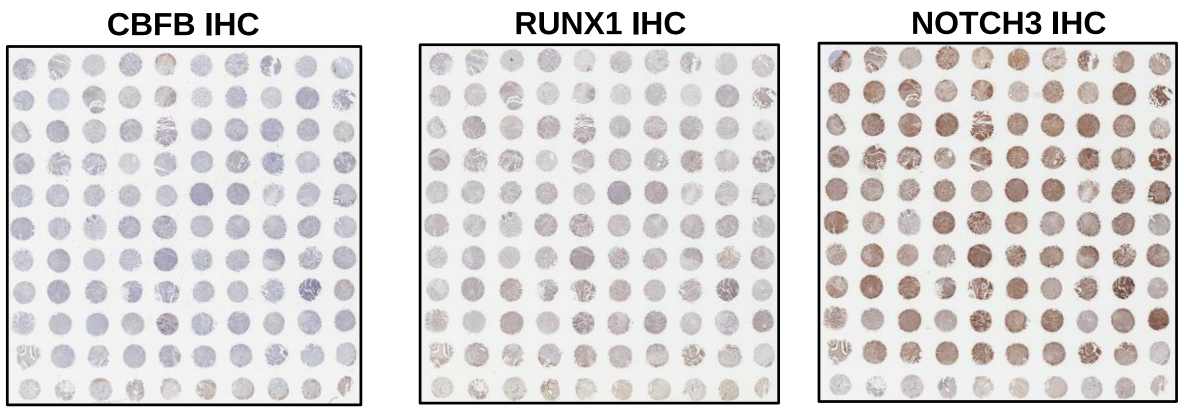

b

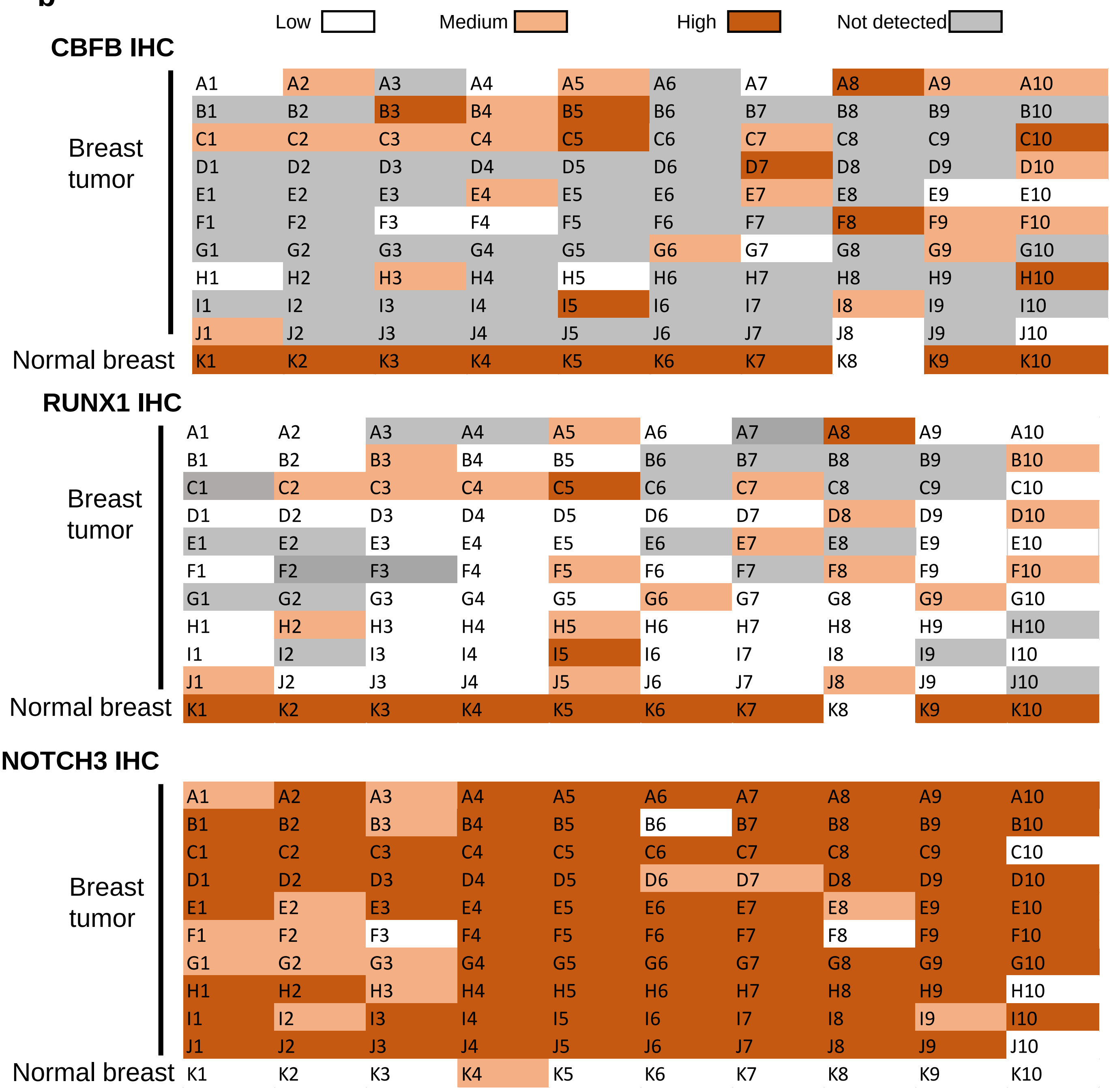

c

Sample type	Protein	Not detected	Low	Medium	High
Normal	CBFB	0/10	1/10	0/10	9/10
	RUNX1	0/10	1/10	0/10	9/10
	NOTCH3	0/10	9/10	1/10	0/10
Tumor	CBFB	69/100	12/100	20/100	9/100
	RUNX1	24/100	53/100	20/100	3/100
	NOTCH3	0/100	5/100	16/100	79/100

Supplementary Fig. 9

d

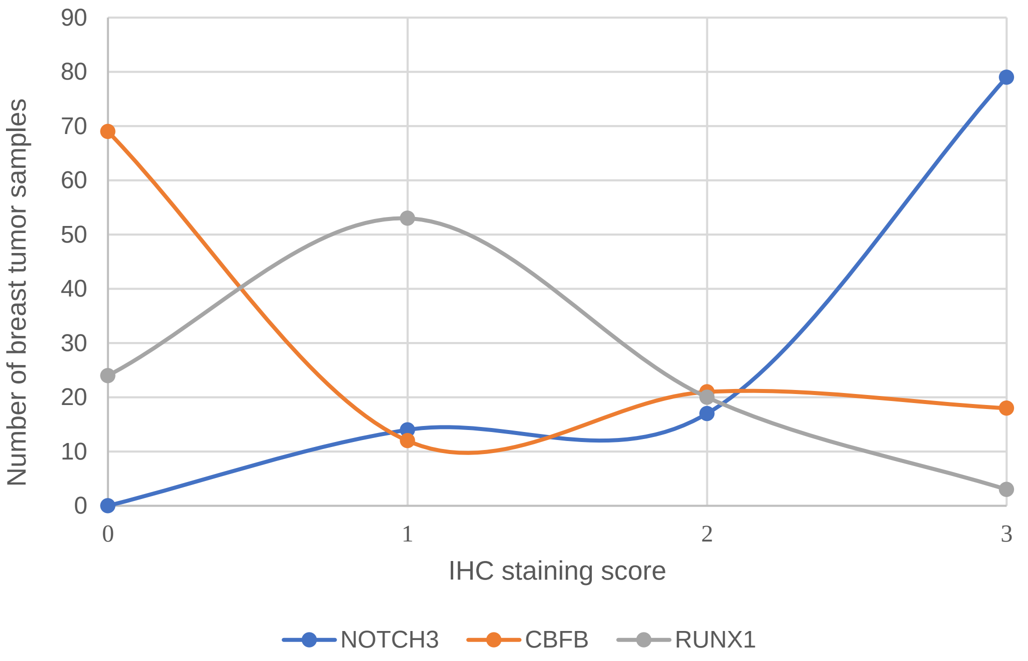

e

	Correlation coefficient (R)	p value
CBFB and RUNX1	0.64	<0.00001
CBFB and NOTCH3	-0.45	<0.00001
RUNX1 and NOTCH3	-0.19	<0.05

Supplementary Fig. 9. CBFB & RUNX1 are downregulated and NOTCH3 is upregulated in human breast tumors. (**a**) CBFB, RUNX1 and NOTCH3 IHC images in human breast cancer tissue microarray (TMA). (**b**) CBFB, RUNX1 and NOTCH3 IHC stain results showing the expression of these proteins in 100 breast cancer cases and 10 normal breast tissue cases in the TMA. A1-J10 are breast cancer tissues and K1-K10 are normal breast tissues. Color codes are intensity of CBFB, RUNX1, and NOTCH3 immunostaining. (**c**) Summary table of CBFB, RUNX1, NOTCH3 IHC staining results. **(d)** Graph displaying IHC staining score of CBFB, RUNX1, NOTCH3 in human breast TMA. Score 0, no signal in staining; score 1, low signal; score 2, medium signal; score 3, high staining. **(e)** Table summarizing the correlation coefficient and p value for CBFB, RUNX1, and NOTCH3 IHC staining signals in human breast TMA.

Genotype	Cell number	Tumor incidence	Latency period (day)	Tumor size (diameter, mm)
WT_Control	5 million	0/4*	No tumor formed 180 days after transplantation	----
CBFB_KO	5 million	5/6	64.5±2.3	12.7±0.51
CBFB_KO + Empty vector	5 million	5/5	68.6±20.9	11.6±2.4
CBFB_KO + CBFB_OE	5 million	0/5	No tumor formed 97 days after transplantation	----
RUNX1_KO	5 million	6/6	62.5±6.9	16±1.15

Supplementary Table 1. Tumorigenicity of different genotypes of MCF10A lines subcutaneously transplanted into NSG mice. WT_control, parental MCF10A cells; CBFB_KO, CBFB knockout MCF10A (clone 751); CBFB_KO + Empty vector, CBFB_KO clone 751 transduced with an empty lentiviral vector; CBFB_KO + CBFB_OE, CBFB_KO clone 751 transduced with a lentiviral vector overexpressing CBFB; RUNX1_KO, RUNX1 knockout MCF10A (clone 5008). Tumor incidence indicates the number of mice having a tumor at euthanizing day versus the total number of mice transplanted. *, one mice died with unknown cause.

Term	PValue	Genes
Integrin signalling pathway	4.64E-04	GRAP, ITGB4, ITGA10, ITGB2, ITGB1, SRC, COL17A1, COL9A2, RAC2, ITGB8, RAC3, COL27A1, ITGAV, COL6A3, ITGB6, RRAS, RHOC, COL8A1, FN1, RAP2B, COL4A4, COL18A1, PIK3C2G, COL13A1, PIK3CD, ITGA1, ITGA3, FLNC, COL5A3, LAMA2, LAMC3, MAPK13, FYN, COL1A1
Alzheimer disease-presenilin pathway	0.003	WNT5A, TRPC1, TCF7, APH1B, MMP19, MMP28, CDH3, LDLRAD3, MMP14, FURIN, MMP2, TCF7L1, MMP25, JUP, NOTCH3, TRIM2, WNT7B, LRP1, PVRL1, LRP10, RNF152, WNT7A
Angiogenesis	0.009	WNT5A, FGFR4, FGFR3, GRAP, JAG1, TCF7L1, SRC, EPHB2, FOS, LPXN, DNER, RRAS, RHOC, ANGPT1, PDGFD, PIK3C2G, ABR, CRYAB, EFNB1, PIK3CD, EFNB2, NOTCH3, EPHA4, SH2D2A, GRB10, WNT7B, SFRP1, MAPK13, GRB7, WNT7A
5-Hydroxytryptamine degredation	0.05	MAOA, ALDH4A1, ALDH1L2, ALDH3B1, ALDH3A1
Axon guidance mediated by semaphorins	0.05	ARHGEF3, NRP1, RAC2, RAC3, FYN, ARHGEF18, DPYSL3, RHOC, NET1
Notch signaling pathway	0.05	NOTCH3, DAB2, MFNG, HES4, DNER, HES2, APH1B, HEY2, JAG1

Supplementary Table 2. Pathway analysis of CBFB-regulated genes at the transcriptional level.

Genotype	Cell number	Tumor incidence	Latency period (day)	Tumor size (diameter, mm)
CBFB Knockout	5 million	4/5	81±0	8±2
CBFB and NOTCH3 double knockout	5 million	0/5	No tumor formed 81 days after transplantation	----
RUNX1 Knockout	5 million	5/5	72.5±12	9.25±5.25
RUNX1 and NOTCH3 double knockout	5 million	0/5	No tumor formed 81 days after transplantation	----

Supplementary Table 3. Tumorigenicity of different genotypes of MCF10A lines subcutaneously transplanted into NSG mice.

Genotype	Cell number	Tumor incidence	Latency period (day)	Tumor size (diameter, mm)
MCF10A empty vector transduced	5 million	0/5	No tumor formed 97 days after transplantation	----
MCF10A Notch3 overexpressing	5 million	5/5	88.6±7.6	10.1±2.1

Supplementary Table 4. Tumorigenicity of different genotypes of MCF10A lines subcutaneously transplanted into NSG mice. MCF10A empty vector transduced indicates MCF10A cells transduced with empty vector; MCF10A Notch3 overexpressing indicates MCF10A cells transduced with a lentiviral vector expressing NOTHC3.
