# Supplementary material for: The transcription factor CBFB suppresses breast cancer through orchestrating translation and transcription
Source: Nat Commun. 2019 May 6;10:2071. doi: 10.1038/s41467-019-10102-6 (PMC6502810; doi:10.1038/s41467-019-10102-6)
Supplement: Supplementary file 3 — Description of Additional Supplementary Files [file 41467_2019_10102_MOESM3_ESM.pdf]

Description of Additional Supplementary Files

File Name: Supplementary Data 1
Description: CBFB interacting proteins identified by mass spectrometry.

File Name: Supplementary Data 2
Description: Oligo information.
