# Supplementary material for: The transcription factor CBFB suppresses breast cancer through orchestrating translation and transcription
Source: Nat Commun. 2019 May 6;10:2071. doi: 10.1038/s41467-019-10102-6 (PMC6502810; doi:10.1038/s41467-019-10102-6)
Supplement: Supplementary file 6 — Reporting Summary [file 41467_2019_10102_MOESM6_ESM.pdf]

nature research

Corresponding author(s): Jing Huang

Reporting Summary

Nature Research wishes to improve the reproducibility of the work that we publish. This form provides structure for consistency and transparency in reporting. For further information on Nature Research policies, see Authors & Referees and the Editorial Policy Checklist.

Statistical parameters

When statistical analyses are reported, confirm that the following items are present in the relevant location (e.g. figure legend, table legend, main text, or Methods section).

n/a	Confirmed	
☐	☒	The exact sample size (n) for each experimental group/condition, given as a discrete number and unit of measurement
☐	☒	An indication of whether measurements were taken from distinct samples or whether the same sample was measured repeatedly
☐	☒	The statistical test(s) used AND whether they are one- or two-sided<br>*Only common tests should be described solely by name; describe more complex techniques in the Methods section.*
☒	☐	A description of all covariates tested
☒	☐	A description of any assumptions or corrections, such as tests of normality and adjustment for multiple comparisons
☐	☒	A full description of the statistics including central tendency (e.g. means) or other basic estimates (e.g. regression coefficient) AND variation (e.g. standard deviation) or associated estimates of uncertainty (e.g. confidence intervals)
☐	☒	For null hypothesis testing, the test statistic (e.g. F, t, r) with confidence intervals, effect sizes, degrees of freedom and P value noted<br>*Give P values as exact values whenever suitable.*
☒	☐	For Bayesian analysis, information on the choice of priors and Markov chain Monte Carlo settings
☒	☐	For hierarchical and complex designs, identification of the appropriate level for tests and full reporting of outcomes
☒	☐	Estimates of effect sizes (e.g. Cohen's d, Pearson's r), indicating how they were calculated
☐	☒	Clearly defined error bars<br>*State explicitly what error bars represent (e.g. SD, SE, CI)*

Our web collection on statistics for biologists may be useful.

Software and code

Policy information about availability of computer code

Data collection	RNA or DNA samples or were sent to NCI next generation sequencing facility to generate libraries and sequenced on HiSeq2500 or NextSeq. Data were collected using standard Illumina protocol and procedure.
Data analysis	We used the public available DESeq algorithm (Love, M. I., Huber, W. & Anders, S. Moderated estimation of fold change and dispersion for RNA-seq data with DESeq2. Genome Biol 15, 550, doi:10.1186/s13059-014-0550-8, 2014) to analyze RNAseq and RIPseq data. For RNAseq, we used FDR=0.05 and fold change at 2 unless specifically indicated in the manuscript. For RIPseq, we used FDR=0.05, fold change at 4 as cutoffs. ChIPseq analysis was done using the algorithm in Zhang et al. Model-based Analysis of ChIP-Seq (MACS). Genome Biol (2008) vol. 9 (9) pp. R137.

For manuscripts utilizing custom algorithms or software that are central to the research but not yet described in published literature, software must be made available to editors/reviewers upon request. We strongly encourage code deposition in a community repository (e.g. GitHub). See the Nature Research guidelines for submitting code & software for further information.

Data

Policy information about availability of data

All manuscripts must include a data availability statement. This statement should provide the following information, where applicable:

- Accession codes, unique identifiers, or web links for publicly available datasets
- A list of figures that have associated raw data
- A description of any restrictions on data availability

Genomic data were submitted to the Gene Expression Omnibus (GEO) database with an accession number: GSE119131 (Polysome profiling), GSE120216 (RNAseq of CBFB WT, KO, RUNX1 WT and KO), GSE119800 (RIPseq of CBFB and hnRNPK), GSE129314 (ChIPseq of RUNX1).

Field-specific reporting

Please select the best fit for your research. If you are not sure, read the appropriate sections before making your selection.

☒ Life sciences ☐ Behavioural & social sciences ☐ Ecological, evolutionary & environmental sciences

For a reference copy of the document with all sections, see nature.com/authors/policies/ReportingSummary-flat.pdf

Life sciences study design

All studies must disclose on these points even when the disclosure is negative.

Sample size	For in vitro experiments, such as RIP, ribosomal profiling, and realtime PCR, we performed three biological repeats. For in vivo experiments, we normally included at least 5 mice for each group. For xenografted experiment using MCF7 cells, we included 10 mice for each group based on previous studies.
Data exclusions	No data were excluded unless the positive control, if available, failed, which indicates experiment failure.
Replication	We took measures to increase the reproducibility of our findings. Replications were successful. 1) We performed three biological replicates for experiments that require statistical analyses. 2) When possible, we used two independent approaches to address the same questions. For example, binding motif of hnRNPK in the RUNX1 mRNA was identified using biochemical approaches and validated with publicly available eCLIP data. RIP data were validated by un-biased RIP-seq data.
Randomization	For in vivo xenograft studies, female mice with same age were randomly assigned to each group.
Blinding	In vitro experiments were not blinding. Xenografting experiment involving MCF7 cells was blind. The person who measured tumor size did not know the genotypes of tumor cells.

Reporting for specific materials, systems and methods

Materials & experimental systems

n/a	Involved in the study	
☐	☒	Unique biological materials
☐	☒	Antibodies
☐	☒	Eukaryotic cell lines
☒	☐	Palaeontology
☐	☒	Animals and other organisms
☒	☐	Human research participants

Methods

n/a	Involved in the study	
☐	☒	ChIP-seq
☒	☐	Flow cytometry
☒	☐	MRI-based neuroimaging

Unique biological materials

Policy information about availability of materials

Obtaining unique materials	In this study, we generated several CRISPR-based knockout of CBFB, RUNX1 and Notch3 in MCF10A cells. These cell lines will be available to the scientific community upon request if shipping cost is provided by requesters.

Antibodies

Antibodies used	For immunoblotting following antibodies were used: p53 (DO1 Santa Cruz, Cat:Sc-126, 1:1000), CBFB (Bethyl, Cat:A303-547A, 1:1000), RUNX1 (Cell signaling, Cat:4334s,1:1000), RUNX2 (cell signaling, Cat:8486 ,1:1000), RUNX3 (Cell signaling, Cat: 9647S ,1:1000), HnRNPK (Bethyl, Cat: A303-674A, 1:1000), NOTCH3 (cell signaling, Cat:5276s , 1:1000), p73 (Abcam, Cat:ab40658, 1:1000), LC3A/B (cell signaling, Cat:12741s, 1:1000), β actin (Sigma, Cat:A5316, 1:5000), H3 (Millipore, Cat:07-690: ,1:5000), GAPDH (Abcam, Cat:ab9484, 1:5000). For Chromatin immunoprecipitation (ChIP), we used following antibodies: p53 (DO1, Santa Cruz, Cat: Sc126, 10 μg) and RUNX1 (Abcam, Cat: Ab23980,10 μg). For Immunohistochemistry (IHC), we used antibodies for CBFB (Bethyl, Cat: A393-549A, 1:100) and RUNX1 (Abcam, Cat: Ab23980,1:100). For Immunofluorescence (IFC), we used following antibodies: CBFB (Bethyl, Cat: A303-549A, 1:1000) and RUNX1(Abcam, Cat: Ab23980,1:1000). For RNA immunoprecipitation (RIP) we used antibodies: CBFB (Bethyl, Cat: A303-549A, 1μg), HnRNPK (Bethyl, Cat: A303-674A, 1μg), and HnRNPL (Bethyl, Cat: A311-423A, 1μg).
Validation	Key antibodies were validated using knockout cell lines or knockdown. For example, CBFB antibody was validated using CRISPR-based knockout cell lines. hnRNPK antibody was validated using siRNA-based knockdown.

Eukaryotic cell lines

Policy information about cell lines

Cell line source(s)	Most cell lines were from American Type Culture collection (ATCC) and MCF7 cell line was from Michael G. Brattain. MCF12A cells were a kind gift from Stefan Ambs (NIH, Bethesda).
Authentication	MCF7 cells were authenticated by short terminal repeat (STR) analysis.
Mycoplasma contamination	All cell lines were tested negative for mycoplasma contamination.
Commonly misidentified lines (See ICLAC register)	Not Applicable

Animals and other organisms

Policy information about studies involving animals; ARRIVE guidelines recommended for reporting animal research

Laboratory animals	NSG mice were from the Jackson Laboratory (JAX,005557, NOD.Cg-Prkdcscid, I12rgtm1Wjl/Szj); athymic nu/nu mice (Nude mice) were from internal breeding at Center for Cancer Research in NCI.
Wild animals	No wild animals were used in this study.
Field-collected samples	*For laboratory work with field-collected samples, describe all relevant parameters such as housing, maintenance, temperature, photoperiod and end-of-experiment protocol OR state that the study did not involve samples collected from the field.*

ChIP-seq

Data deposition

☒ Confirm that both raw and final processed data have been deposited in a public database such as GEO.

☒ Confirm that you have deposited or provided access to graph files (e.g. BED files) for the called peaks.

Data access links *May remain private before publication.*	https://www.ncbi.nlm.nih.gov/geo/query/acc.cgi?acc=GSE129314
Files in database submission	GSM3704447 for MCF10A cells_Input DNA, GSM3704448 for MCF10A cells_RUNX1 ChIPseq
Genome browser session (e.g. UCSC)	no longer applicable

Methodology

Replicates	1 repeat
Sequencing depth	GSM3704447, total reads, 96132262, uniquely mapped reads, 69818605<br>GSM3704448, total reads, 91726821, uniquely mapped reads, 66238355
Antibodies	RUNX1 (Abcam, Cat: Ab23980,10 μg)
Peak calling parameters	callpeak -t CHNM36.bam.sorted.bam -c CHNM35.bam.sorted.bam -f BAM -n RUNX1ChIP_MCF10AOEq0.05 -g hs -B -q 0.05
Data quality	Using the peak calling parameters above, we have detected about 13,000 peaks. The data quality is only average compared

Data quality	to the ChIPseqs performed in our laboratory. The main reason could be either that our RUNX1 antibody (the best among the several tested) has low affinity (technical reason) and/or that RUNX1 does not bind to chromatin strongly as majority of its binding partner CBFB is in the cytoplasm. Previous studies demonstrated that CBFB enhances RUNX1 chromatin binding (biological reason).
Software	We used published the MACS algorithm described in Y. Zhang, T. Liu, C. A. Meyer, J. Eeckhoute, D. S. Johnson, B. E. Bernstein, C. Nusbaum, R. M. Myers, M. Brown, W. Li and X. S. Liu. Model-based Analysis of ChIP-Seq (MACS) Genome Biology 2008.
